# Integrated morphophysiological, transcriptomic, and metabolomic data uncover the molecular mechanism of environmental adaptation of *Zanthoxylum armatum* with different latitudinal gradients

**DOI:** 10.3389/fpls.2025.1622956

**Published:** 2025-07-01

**Authors:** Yuan Guo, Xueqian Fu, Chong Sun, Yifei Deng, Han Liu, Long Tong, Mi Kuang, Ning Tang, Wenying Yang, Xia Liu, Zexiong Chen

**Affiliations:** ^1^ Chongqing Key Laboratory of Economic Plant Biotechnology/Collaborative Innovation Center of Special Plant Industry in Chongqing/College of Smart Agriculture, Chongqing University of Arts and Sciences, Chongqing, China; ^2^ Hubei Key Laboratory of Spices & Horticultural Plant Germplasm Innovation and Utilization, Yangtze University, Jingzhou, China; ^3^ Bamboo Research Institute, Chongqing Academy of Forestry, Chongqing, China; ^4^ Chongqing Agricultural Technology Promotion General Station, Chongqing, China; ^5^ Rongchang District Forestry Science and Technology Extension Station of Chongqing, Chongqing, China

**Keywords:** *Zanthoxylum armatum*, transcriptome, metabolome, RT-qPCR, environmental adaptability

## Abstract

**Introduction:**

Leaves are sensitive to environmental changes and directly reflect the degree of environmental impact on plants and their ability to adapt to the environment, making it crucial to understand the genetic mechanisms underlying leaf variation. *Zanthoxylum armatum* is a widely distributed and economically important forest species in China that shows remarkable regional adaptability. However, adaptive differences under diverse environmental conditions and their molecular mechanisms have not been systematically studied.

**Methods:**

Plant materials of *Z. armatum* from three regions (Shandong, Chongqing, and Yunnan) representing different latitudinal backgrounds were cultivated under uniform conditions. Morphological, physiological, and biochemical traits were measured, including stomatal density, nutrient content, antioxidant capacity, and chlorophyll level. Transcriptomic and metabolomic profiling were conducted using RNA-seq and UPLC-MS/MS, respectively. Differential expression and enrichment analyses (GO, KEGG), gene family screening, and correlation analyses were used to identify key genes and metabolites. Selected gene expression patterns were further validated using qRT-PCR.

**Results:**

Under common garden conditions, the three *Z. armatum* populations retained distinct physiological and molecular profiles. SD, CQ, and YN groups showed respective advantages in antioxidant activity, nutrient accumulation, and chlorophyll content. Integrated transcriptomic and metabolomic analyses identified seven resistance-related and two photosynthesis-associated genes, significantly correlated with physiological traits. Ninety-two differential metabolites were detected, including two enriched in phenylpropanoid and flavonoid pathways. The YN group exhibited more coordinated gene expression across key metabolic pathways, indicating greater potential for metabolic flux. These results highlight molecular features underlying population-level variation under common garden.

**Discussion:**

Through multi-level comprehensive research, a new perspective has been provided for revealing the molecular regulatory network of the environmental adaptability of *Z. armatum*. In the future, we can use plant genome editing tools to target these genes as the bases and transform them into *Z. armatum* varieties with multiple resistance qualities, thereby contributing to scientific research and commercial Sichuan pepper cultivation.

## 1 Introduction

Plant growth and distribution are strongly limited by environmental conditions, and ecological differences in various regions pose challenges to plant adaptability. Plants work synergistically to adapt to different growth environments through complex molecular regulatory networks, including physiological characterization optimization, transcriptome responses, and metabolic regulation (Zhu, 2016; Avila et al., 2023). Environmental adaptability is the core ability of plants to survive and reproduce in various ecosystems. Intraspecific variation has been recognized as a key component of plant response strategies to diverse environmental backgrounds, and may reflect either heritable differentiation or phenotypic plasticity (Henn et al., 2018). Understanding the genetic and physiological mechanisms underlying plant adaptability is of great significance for understanding environmental adaptability and promoting resource optimization.

Previous studies have shown that plants can enhance their adaptability to diverse environmental conditions through synergistic regulation at the transcriptome and metabolome levels (Dong and Lin, 2021; Song et al., 2024). Transcriptome analysis based on RNA-seq technology has revealed that when plants respond to drought or high-temperature stress, the expression of antioxidant enzyme genes (such as superoxide dismutase and catalase) is usually significantly up-regulated, which helps reduce oxidative damage and contributes to the regulation of internal physiological processes (Wang and Cheng, 2017; Santos et al., 2023). Changes in the accumulation of primary and secondary metabolites have been shown to modulate plant physiological responses under varying environmental factors, including osmotic and oxidative challenges (Zhang et al., 2023; Kumar et al., 2021). At the morphophysiological level, plants can regulate the synthesis of secondary metabolites, such as phenols (Nicholson and Hammerschmidt, 1992), flavonoids (Ramaroson et al., 2022), and terpenoids (Li et al., 2023), and dynamic changes in chlorophyll content (Cutolo et al., 2023), which together help maintain metabolic homeostasis and support photosynthetic performance under changing conditions. These metabolites not only enhance the antioxidant capacity and UV protection ability of plants but also play a role in maintaining overall metabolic balance. It is worth noting that secondary metabolites such as phenols, flavonoids, and terpenoids are not only key components of plant physiological regulation, but their accumulation levels are also closely related to the flavor quality and medicinal activity of crops (Xu et al., 2023b; Yang et al., 2023). Besides these, other studies have also suggested that plants from different geographic origins may exhibit significantly different molecular characteristics, even when grown under identical conditions (Monteiro et al., 2019; Wu et al., 2018). This indicates that long-term environmental heterogeneity may contribute to such baseline differences among populations.

*Zanthoxylum armatum* DC. is a species in the Rutaceae family, widely used by consumers both domestically and internationally for its unique flavor and medicinal value, and its strong adaptability enables it to grow in a variety of complex environments (Hu et al., 2023; Liu et al., 2023a; Hui et al., 2022). The natural population of *Z. armatum* shows abundant leaf shape variation. However, differences in environmental adaptation and the related molecular mechanisms under different environmental conditions have not been systematically studied. Therefore, three representative regions with significant latitudinal gradient differences were selected for the current study: Shandong Province (high latitude, warm temperate monsoon climate), Chongqing Municipality (mid latitude, subtropical humid monsoon climate), and Yunnan Province (low latitude, subtropical plateau monsoon climate, with some areas exhibiting a tropical monsoon climate). Leaf samples of *Z. armatum* were collected from a common garden. Morphological, physiological, biochemical, transcriptomic, and metabolic data were used to systematically analyze the phenotypes and molecular differences of *Z. armatum* in different ecological environments, as well as the synergistic effects of key genes and metabolites to reveal how *Z. armatum* can adapt to diverse environmental conditions. The results not only provide a theoretical basis for understanding the environmental adaptability of forest trees but also provide valuable genetic resources for the improvement of *Z. armatum* cultivars.

## 2 Materials and methods

### 2.1 Materials

In 2022, wild seedlings of *Z. armatum* distributed in three latitudinal regions, Shandong Province (SD; N34°58′59″, E117°17′41″), Chongqing City (CQ; N29°12′50″, E105°50′47″), and Yunnan Province (YN; N25°39′54″, E101°54′25″), were collected and planted in the same germplasm nursery (common garden). The plant materials grew for one year under unified management conditions in the common garden (N29°10′44″, E105°49′59″). On June 25, 2023, mature leaves of *Z. armatum* in the middle part of the new shoots were used as the material, with three biological replicates for each sample. After collection, the leaves were placed in 50 mL centrifuge tubes, immediately frozen in liquid nitrogen, transported to the laboratory, and stored at -80°C for further analysis.

### 2.2 Observation of stomatal density in leaves

Healthy leaves of *Z. armatum* were selected and observed under a confocal laser-scanning microscope (CLSM; LSM 700, ZEISS, Oberkochen, Germany). Small leaf sections were fixed with the abaxial side facing upward, covered with glycerol, and examined. The morphology and distribution of stomata were recorded using a 488 nm laser and detection light with a wavelength range of 500–550 nm. High-resolution images were acquired using ZEISS ZEN software and the key parameters of the stomata were analyzed. For each sample, take three random photos and randomly select ten fields of view from each image for stomata counting. One-way analysis of variance (ANOVA) was conducted on the statistical data using GraphPad Prism v9.0, to evaluate significant differences among the three groups.

### 2.3 Physiological and biochemical index measurements

A series of physiological indicators, including total carbon, nitrogen, phosphorus, chlorophyll, and amino acid contents, were measured. The determination of total carbon content was conducted in accordance with the potassium dichromate titration method described by Liu et al. (2023b). Total nitrogen (N) and phosphorus (P) contents were determined using the Kjeldahl method and molybdenum-antimony-blue spectrophotometry, respectively, based on acid-digested oven-dried samples, with results expressed as g/100 g dry weight (Bradstreet, 1954; Jiao et al., 2024). Chlorophyll content was measured using fresh samples extracted with a buffered reagent under dark conditions, with absorbance values recorded at 649 nm and 665 nm. Chlorophyll a, chlorophyll b, and total chlorophyll contents were calculated using the Lambert-Beer law and expressed as mg/g fresh weight. Amino acid content was determined using the ninhydrin colorimetric method, based on fresh samples extracted with a specific buffer at room temperature, and expressed as μmol/g fresh weight (Yang et al., 2013).

A series of biochemical indicators, including FRAP, ABTS, DPPH, total flavonoids, total phenols, total terpenoids, and alkaloids, were measured. The total antioxidant capacity was assessed using three assays: ferric reducing antioxidant power (FRAP), 2,2´-azino-bis(3-ethylbenzothiazoline-6-sulfonic acid) (ABTS), and 2,2´-diphenyl-1-picrylhydrazyl-hydrate (DPPH). In the FRAP assay, antioxidants reduce Fe³^+^-TPTZ to form a blue Fe²^+^-TPTZ complex in an acidic environment, with absorbance measured at 590 nm. The ABTS^+^ radical cation decolorization method measured absorbance at 734 nm, while the DPPH assay quantified radical scavenging activity by detecting the decrease in absorbance at 517 nm (Dudonné et al., 2009). Samples were extracted using suitable buffers or solvents. FRAP reagent was prepared from sodium acetate buffer (pH 3.6), FeCl₃, and TPTZ. ABTS radical cation was generated from ABTS and potassium persulfate and diluted to ~0.70 absorbance. DPPH reagent was a 0.1 mmol/L methanol solution. Total phenol and flavonoid contents were determined using the Folin-Ciocalteu and NaNO_2_-Al(NO₃)₃-NaOH colorimetric methods, respectively, based on fresh samples extracted with 60% ethanol at 60°C, and expressed as mg/g fresh weight (Jia et al., 2015; Zhu et al., 2024). Total terpenoid and alkaloid contents were measured using the vanillin-perchloric acid colorimetric method and the Plant Alkaloid Content Detection ELISA Kit (Shanghai Kanglang Biotechnology Co., LTD, China), respectively, both based on oven-dried samples extracted with chloroform (for terpenoids) and 80% methanol at 60°C (for alkaloids), and expressed as mg/g dry weight (Jiang et al., 2016). All calculation formulas are provided in **Supplementary Table S1**.

### 2.4 RNA sequencing and data processing

Total RNA was extracted using TRIzol reagent, RNA integrity was assessed using an Agilent 2100 Bioanalyzer (Agilent Technologies, Palo Alto, CA, USA), and RNA purity was measured using a NanoDrop ND-2000 (Thermo Fisher Scientific Inc., USA) to ensure that sample quality met library construction requirements. Next, mRNA was enriched using the oligo (dT) method, fragmented, and reverse transcribed to cDNA. Adapters were added, followed by PCR amplification to construct a sequencing library. After concentration and insert size detection, the library was quantified using a TBS380 fluorometer and subjected to paired-end 150 bp high-throughput sequencing on an Illumina platform to generate raw reads. Raw reads were processed to remove adapters and low-quality sequences, resulting in clean reads. The clean reads were then aligned to the *Z. armatum* reference genome (Hu et al., 2023) using HISAT2 (Kim et al., 2019), and alignment rates and distribution were analyzed. The transcripts were assembled using StringTie and compared with reference transcripts using Cuffcompare to identify novel genes (Trapnell et al., 2012). The expression level of each transcript was calculated based on normalized expression (fragments per kilobase per million mapped reads, FPKM). Gene abundance was quantified using RSEM (Li and Dewey, 2011). novel and existing genes were annotated by alignment with the NR, Swiss-Prot, GO, eggNOG, COG, KOG, KEGG, and Pfam databases using BLAST.

### 2.5 Transcriptome variation and functional analysis

Alternative splicing, SNP variation, and differential gene expression analyses were performed to further explore transcriptomic differences in *Z. armatum*. Alternative splicing events were identified using rMATS (Shen et al., 2014), which calculated the expression ratio of each splicing type and performed statistical testing. *P*-values were adjusted using false discovery rate (FDR) correction to assess significant differences among groups. To investigate genomic variation, SNPs and InDels were identified from RNA-seq data. Clean reads were aligned to the reference genome using HISAT2 to generate BAM files, and variant calling was performed using GATK (Brouard et al., 2019) after removing PCR duplicates. Preliminary SNP and InDel sites were then obtained. Differential expression analysis was conducted using the DESeq2 package in R, which normalized raw count data using the median-of-ratios method to account for differences in sequencing depth and other technical factors. A model based on the negative binomial distribution was applied to perform pairwise comparisons among the three sample groups. Genes were considered differentially expressed if they met the criteria: *P*-value < 0.05, FDR < 0.05, and |log_2_FoldChange| ≥ 1. To functionally interpret the DEGs, Gene Ontology (GO) and Kyoto Encyclopedia of Genes and Genomes (KEGG) enrichment analyses were performed using an online platform (https://www.omicshare.com/tools/). Enrichment was evaluated using hypergeometric testing, and *P*-values were corrected by FDR. Pathways or GO terms with *P*-values (FDR ≤ 0.05) were considered significantly enriched and were visualized accordingly.

### 2.6 Metabolomics sequencing and differential metabolites screening

Metabolites were detected using a Shimadzu UFLC UPLC system coupled to a Sciex QTRAP^®^ 4500+ mass spectrometer (Shim-pack CBM A system, Waters HSS T3 C18 column, 1.8 µm, 2.1×100 mm), with three biological replicates per sample. Raw MS data were processed using Analyst 1.6.3, and peak areas were integrated using MultiaQuant software to generate a sample–metabolite peak area matrix. The matrix was log_2_-transformed and normalized using unit variance scaling prior to statistical analysis. Orthogonal partial least squares discriminant analysis (OPLS-DA) combined with orthogonal signal correction (OSC) was used to conduct an in-depth analysis of the metabolites between the sample groups. Data analysis was performed using the MetaboAnalystR package in the R software. Based on the OPLS-DA results, differential metabolites were screened according to the following criteria: fold change ≥ 2 or fold change ≤ 0.5, and a variable importance in projection (VIP) value ≥ 1. Furthermore, this study screened for metabolites that were significantly differentially expressed across all three sample groups and annotated them in the KEGG database to explore the potential metabolic pathways involved.

### 2.7 Gene family identification and correlation analysis

The functional domains and gene families of the identified key genes were analyzed using the Pfam database (http://pfam-legacy.xfam.org/). Gene families related to resistance and photosynthesis, such as the light-harvesting complex (LHC; PF00504) and glutathione peroxidase (GPX; PF00255), were selected and screened using HMMER3.0, with an E-value < 0.05. The structural domains of the candidate genes were checked using the Conserved Domain Database from the NCBI (https://www.ncbi.nlm.nih.gov/cdd/). Intersection analysis was performed between the genes selected by HMMER and the differentially expressed genes to identify the key genes expressed across multiple groups. Subsequently, correlation analysis was performed using an online tool (https://www.omicshare.com/tools/). Pearson correlation coefficients were calculated to assess the relationships between differentially expressed genes and physiological indices, and between differential metabolites and physiological indices.

### 2.8 qRT-PCR validation analysis of key candidate genes

Three candidate reference genes, UBQ, EF2-4, and GAPDH, were initially selected for evaluation (Fei et al., 2018; Fan et al., 2024; Zheng et al., 2025). Their expression stability in *Z. armatum* leaves was assessed using RefFinder (Xie et al., 2023), an integrative tool combining geNorm (Vandesompele et al., 2002) and BestKeeper (Pfaffl et al., 2004). Based on the results, UBQ was selected as the most stable internal control gene for normalization (**Supplementary Figure S3**). Specific primers were designed using Primer 5.0 software, with forward (F) and reverse (R) primers having melting temperatures between 55–60°C and differences within 3°C. Amplification products were controlled to 120–180 bp in length, and primer specificity was confirmed via BLAST (https://www.ncbi.nlm.nih.gov/). Total RNA was extracted using the Tiangen RNA Prep Pure Polysaccharide Plant Total RNA Extraction Kit (Tiangen Biotech (Beijing) Co., Ltd., China), and reverse transcription was performed using the HiScript III RT SuperMix for qPCR (+ g DNA wiper) kit (Nanjing Vazyme Biotech Co. Ltd., Nanjing, China). qRT-PCR reactions were run in 20 μL volumes on a qTOWER2.2 fluorescence quantitative PCR system. Three biological replicates were used, and relative gene expression levels were calculated by the 2^-ΔΔCT^ method (Livak and Schmittgen, 2001).

### 2.9 Statistical analysis

Data were analyzed for statistical significance using Excel 2020 and SPSS software 20.0, with a one-way ANOVA and Duncan’s test (*P* < 0.05) being used to determine the significance of differences. GraphPad Prism 9 was used to plot the data.

## 3 Results

### 3.1 Phenotypic variation and physiological–biochemical indicators of *Zanthoxylum armatum*

**Figure 1:**
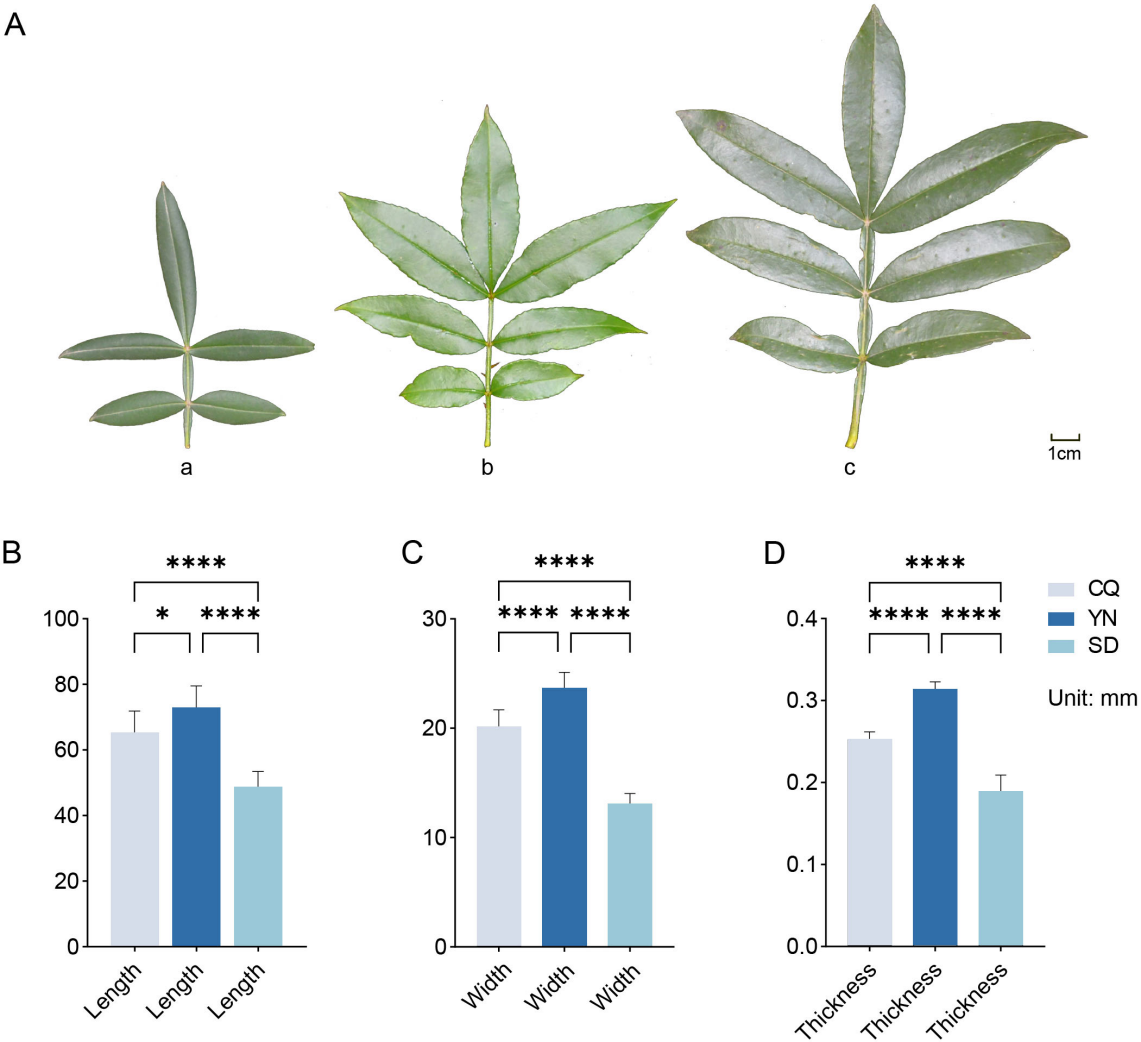
Morphology of *Z. armatum* leaves and phenotypic data. **(A)** Leaves of *Z. armatum* from the SD, CQ, and YN province, with **(a–c)** showing the front side of the leaves. **(B–D)** Phenotypic data, including length, width, and thickness. *P<0.05, ****P<0.0001.

There were significant differences in leaf size, morphology, and leaf axis characteristics among the three groups (**Figure 1A**). Measurements of 10 leaf samples per group (**Figures 1B–D**; **Supplementary Table S2**) revealed that plants in all three groups had 5–7 leaves. The leaves of plants in the SD group were smaller and narrower, with a slightly waxy surface. The average length, width, and thickness of the leaves were 48.74 mm, 13.08 mm, and 0.19 mm, respectively, and they were arranged in an opposite arrangement. The leaves of the CQ group were broader than those of the SD group and retained a relatively narrow and elongated shape. The average length, width, and thickness were 65.32 mm, 20.14 mm, and 0.25 mm, respectively, with a distinct petiole structure and a spiny surface, which may serve as a physical barrier. The leaves of the YN group were the largest with prominent oil cells and elongated shapes. The average leaf length, width, and thickness were 72.95 mm, 23.66 mm, and 0.31 mm, respectively. Compared with the CQ group, the YN and SD groups exhibited distinct compound leaf structures in the petiole.

**Figure 2:**
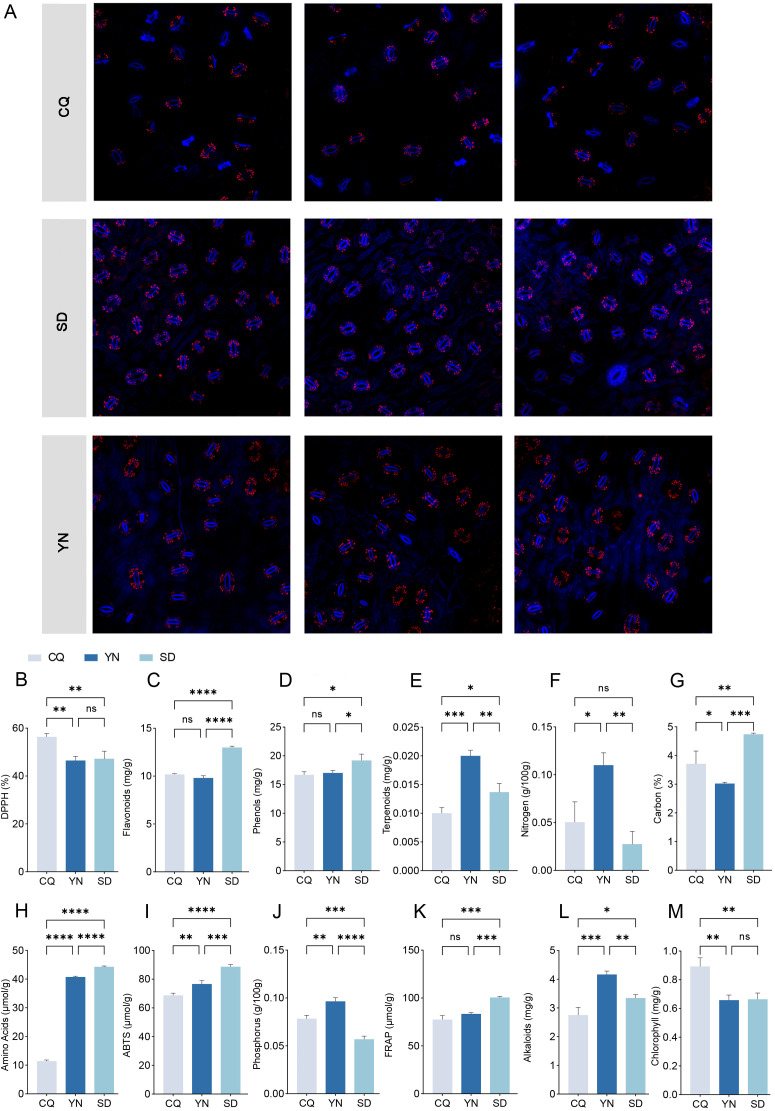
**(A)** Laser confocal microscopy images of leaf stomata in SD, CQ and YN groups. Three replicates per group. Stomata are shown in blue. **(B-M)** Physiological indicators. Data are presented as the mean ± SEM. Statistical significance was determined using ANOVA for multiple-group comparisons. *P<0.05, **P<0.01, ***P<0.001, ****P<0.0001.

Physiological and biochemical indicator analysis (**Figures 2B–M**; **Supplementary Table S3**) showed that the three groups exhibited distinct advantages in terms of antioxidant, defense, and photosynthesis-related indices. The SD group showed the highest levels of flavonoids and phenols, as well as the strongest antioxidant activity according to the FRAP and ABTS assays. Compared to the SD group, the YN group had superior N and P contents, with relatively higher accumulation of alkaloids and terpenoids, while the antioxidant capacity of the YN group (FRAP and ABTS assays) was slightly lower than that of the SD group and remained relatively high, with flavonoid and phenol contents close to that of the SD group. The CQ group showed higher chlorophyll content than the SD and YN groups. Although its flavonoid and phenol contents were slightly lower than those of the other two groups, its DPPH value was higher.

### 3.2 Leaf stomata characteristics

To further explore the environmental adaptation mechanisms of *Z. armatum*, this study examined the stomatal characteristics of plant leaves in the SD, CQ, and YN groups using CLSM (**Figure 2A**). Each group comprised three biological replicates. In an observation field of 320 × 320 μm, the average number of stomata was approximately 37, 24, and 30 in the SD, CQ, and YN groups, respectively. The stomatal density was 365.63/mm², 234.38/mm², and 296.88/mm² in the SD, CQ, and YN groups, respectively. The stomatal density in the SD group was significantly higher than that in the CQ and YN groups.

### 3.3 Variable clipping and SNP difference analysis of transcriptome data

Transcriptome sequencing results showed that the total number of reads ranged from 37,665,298 to 136,607,856 and the proportion of clean reads exceeded 99% in all samples. The GC content ranged from 43.30% to 43.86%, and the Q20 and Q30 percentages were 98.01–98.15% and 93.78–94.24%, respectively (**Supplementary Table S4**). These results indicated high sequencing accuracy and stability. The mapping rate for all samples exceeded 86%, indicating good alignment with the reference genome and ensuring data suitability for subsequent gene expression analyses.

**Figure 3:**
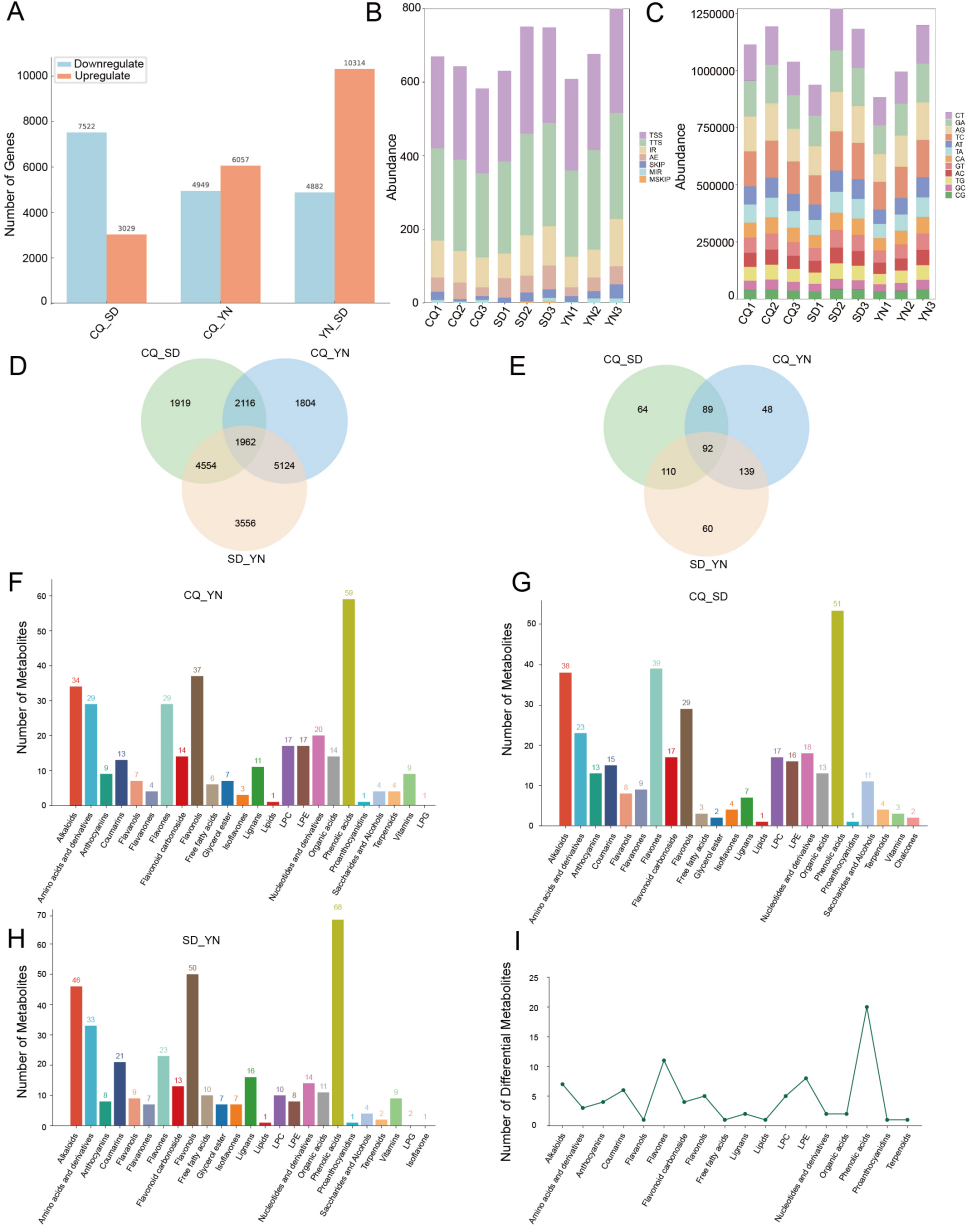
**(A)** The transcriptome differential analysis plot, displaying pairwise comparisons between the three regions, with blue indicating downregulated genes and orange indicating upregulated genes. **(B)** A stacked bar plot of alternative splicing events, where different colors represent different types of alternative splicing events. **(C)** A stacked bar plot of SNPs, with colors indicating the number of base substitutions. The x-axis represents different groups, and the y-axis represents the specific counts. **(D)** Venn diagram of differentially expressed genes between groups. **(E)** Venn diagram of differentially abundant metabolites between groups. **(F–H)** show the number of differential metabolites between CQ、YN and SD, with different colors representing different metabolites. **(I)** Shows the different types of metabolite categories and their quantities detected among the differential metabolites.

To reveal differences in gene expression in *Z. armatum*, alternative splicing events in the samples were analyzed, and the average splicing event frequencies for the CQ, SD, and YN groups were calculated (**Figure 3B**; **Supplementary Table S5**). The results revealed significant differences in the expression of different splicing events in the samples. The SD group showed significantly higher frequencies of transcription start site (TSS), transcription termination site (TTS), multi-exon skipping (MSKIP), and alternative exon ends (AE) events compared to the CQ and YN groups, reflecting a broader range of splicing event types. The YN group had the highest frequencies of single exon skipping (SKIP), multiple intron retention (MIR), and intron retention (IR) events but lacked MSKIP events, with exon-related splicing events more frequently detected. In contrast, the CQ group had the lowest frequency of most splicing events, except for a slightly higher frequency of MIR events than the SD group. No MSKIP events were detected, and the overall splicing frequency was relatively low.

Based on the SNP analysis results (**Figure 3C**; **Supplementary Table S6**), the average frequencies of base substitutions were calculated. The SD group had the highest frequency of all substitution types, except for CG and GC, where the SD group which ranked second. The CQ group had the highest frequency of CG and GC substitutions, whereas it ranked second for all other substitution types. In contrast, the YN group had the lowest frequency of all substitution types. These results indicated differences in base substitution patterns among the three groups, with the SD group showing a generally higher substitution frequency, the CQ group showing elevated CG and GC substitution frequencies, and the YN group exhibiting consistently low substitution levels.

### 3.4 Metabolomics analysis

**Figure 4:**
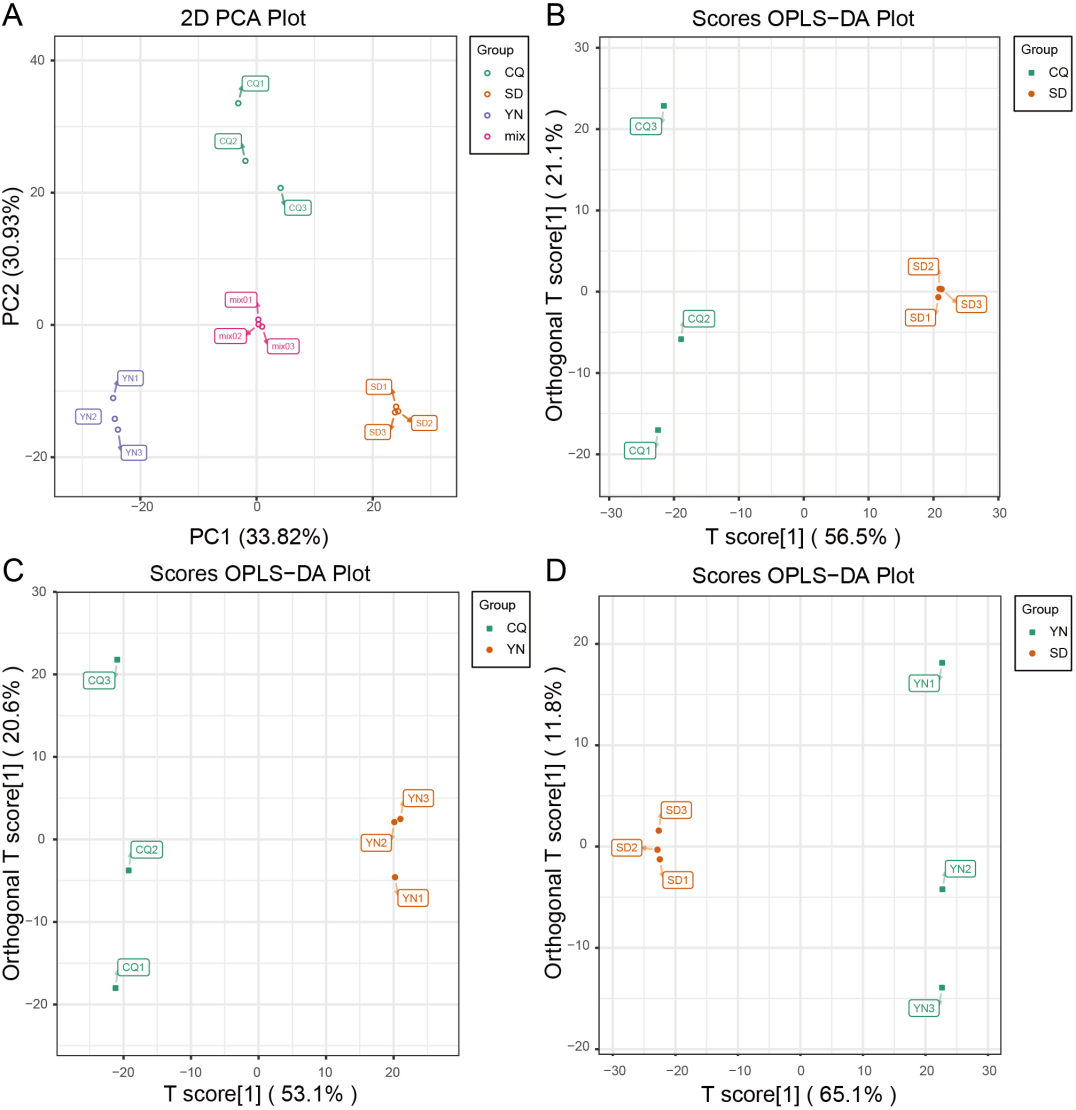
Principal component analysis. **(A)** PCA plot, with PC1 on the x-axis and PC2 on the y-axis. **(B)**, **(C)**, and **(D)** OPLS-DA score plots with T score [1] on the x-axis and T score [2] on the y-axis. Green represents CQ, purple represents YN, orange represents SD, and pink represents Mix.

Metabolite detection was performed using the Shimadzu-Sciex UPLC-MS/MS platform. In total, 953 metabolites were identified, covering 26 categories (**Supplementary Table S7**). The results of principal component analysis (PCA, **Figure 4A**) showed a clear separation between the SD, CQ, and YN groups, with significant intergroup differences and relatively tight intragroup sample distributions. The mixed sample (Mix), used as a quality control, was located centrally between the groups, further validating the reliability of the metabolomic data. These results indicate regional specificity in the metabolic composition of *Z. armatum* in different regions. PC1 and PC2 explained 33.82% and 30.93% of the metabolic variation, respectively, indicating that the principal components could be effectively distinguished between the sample groups. The distinct clustering of the groups in the principal component space further supported the metabolic differences, which were consistent with their geographic origins, as revealed by PCA.

### 3.5 Differential analysis of transcriptome and metabolome data

Differential expression analysis of the transcriptome data was performed using DEseq2 software (**Figure 3A**), with a significance threshold of *P* < 0.05 and FDR < 0.05, and a differential expression criterion of |log2FoldChange| ≥ 1. The results showed that in the CQ-SD comparison, 10,551 differentially expressed genes were identified (**Supplementary Table S8**), including 80 novel genes. Of these, 7,522 were downregulated and 3,029 were upregulated. In the CQ-YN comparison, 11,006 differentially expressed genes were identified (**Supplementary Table S9**), including 103 novel genes with differences. Among these, 4,949 were downregulated and 6,057 were upregulated. In the YN-SD comparison, 15,196 differentially expressed genes were identified (**Supplementary Table S10**), including 113 novel genes. Of these, 4,882 were downregulated and 10,314 were upregulated. Intersection analysis of the three comparisons revealed 1,962 differentially expressed genes that exhibited significant differential expression across all three comparisons (**Figure 3D**).

For metabolome data, OPLS-DA combined with OSC was used for the pairwise differential analysis of metabolites among the three *Z. armatum* groups (SD, CQ, and YN) (**Figures 3F–H**, **4B–D**; **Supplementary Tables S11**-**13**). The results showed a significant separation in the metabolite expression patterns among the CQ-SD, CQ-YN, and YN-SD groups, further validating the reliability of the OPLS-DA model. To evaluate the stability and predictive ability of the model, 200 permutation tests (**Supplementary Figure S1**). The results based on R²X, R²Y, Q², and P-values indicated that the model had high stability and good predictive capability, with no signs of overfitting. Based on the screening criteria of VIP values ≥ 1 and fold change ≥ 2 or ≤ 0.5, we identified 92 significantly different metabolites consistently present across the three groups (**Figure 3E**, **3I**; **Supplementary Table S14**). These metabolites might play important roles in the metabolic regulation of the three *Z. armatum* groups, thereby providing a critical basis for subsequent analyses.

### 3.6 Integrative transcriptomic and metabolomic analysis reveals functional and expression differences in oxidative metabolism pathways among populations

**Figure 5:**
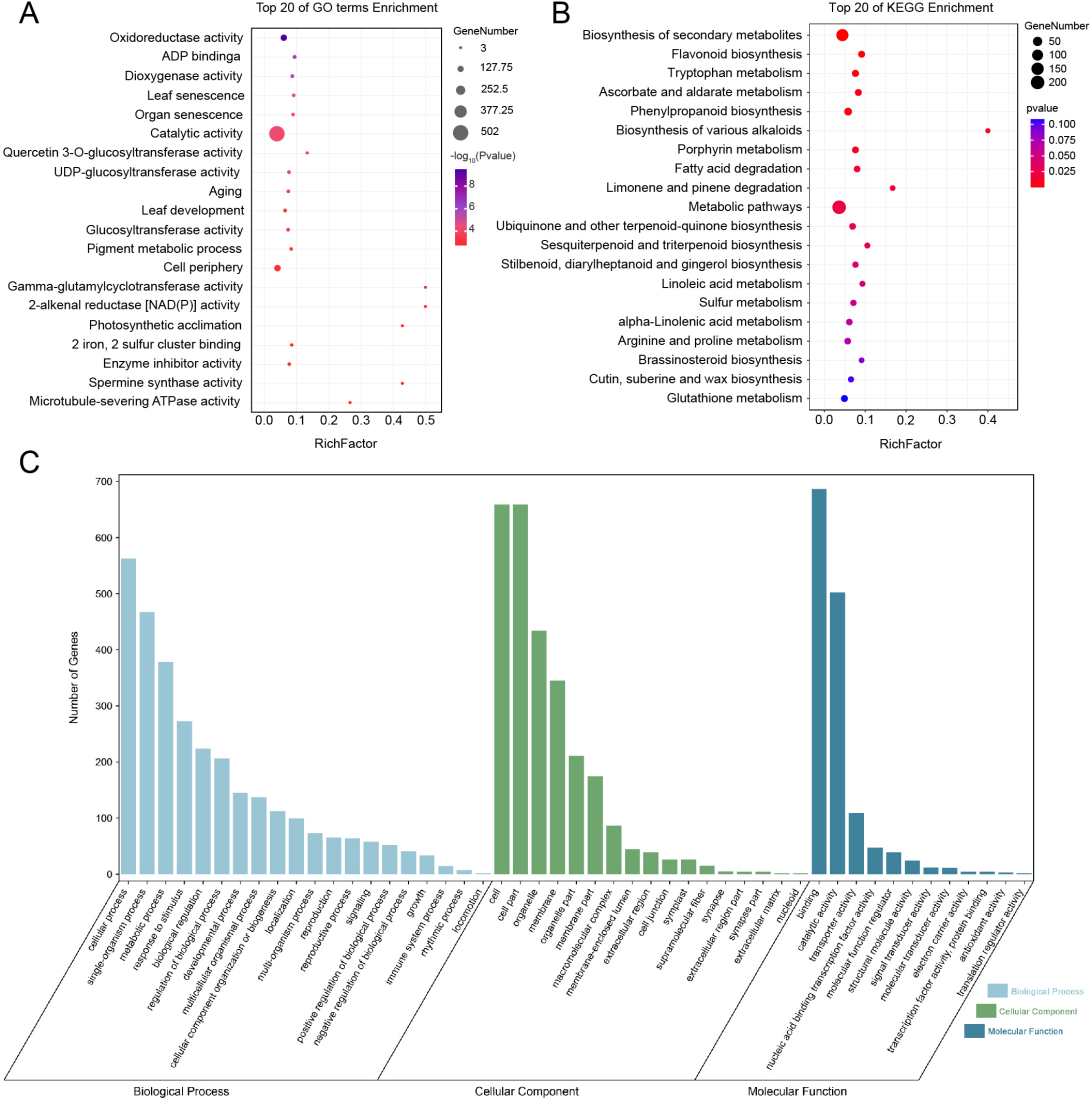
GO and KEGG enrichment analyses. **(A)** The top 20 enriched pathways from the GO enrichment analysis, with bubble size representing gene count and color indicating significance (red = significant, blue = non-significant, -log10 of P-value). **(B)** The top 20 enriched pathways from the KEGG analysis, with similar bubble size and color coding, using a P-value threshold of < 0.05. **(C)** with different colors representing different categories: light blue for Biological Process, green for Cellular Component, and dark blue for Molecular Function. The degree of enrichment is represented by a bar chart.

To investigate the molecular basis underlying group-level differences in *Z. armatum*, we first performed GO enrichment analysis on 1,962 differentially expressed genes (DEGs) (**Figures 5A–C**). Oxidoreductase activity was identified as the most significantly enriched functional category, comprising 131 DEGs. As this category is closely associated with antioxidant processes and redox regulation, we further examined specific gene families involved in these functions. Based on the GO results, we conducted a genome-wide screening of the glutathione peroxidase (GPX) gene family, which encodes key antioxidant enzymes that mitigate oxidative stress by reducing hydrogen peroxide and lipid hydroperoxides (Pei et al., 2023). A total of 16 GPX candidate genes were identified (E-value < 0.05; **Supplementary Table S15**), and one of which, *ZaGPX1* (*ZaA2_C21.Contig2153.4-gene*) overlapped with the DEGs. To further explore functional pathways related to redox regulation, KEGG enrichment analysis was performed using both the DEGs and 92 differentially expressed metabolites (DEMs). These metabolites were selected from those consistently identified as significantly different across all three group comparisons (**Figure 3E**, **Supplementary Table S14**). Consistent with the GO findings, phenylpropanoid biosynthesis (ko00940) and flavonoid biosynthesis (ko00941) emerged as significantly enriched pathways (*P* < 0.01). Both contribute to oxidative stress mitigation via the synthesis of phenolic acids, flavonoids, and lignin.

**Figure 6:**
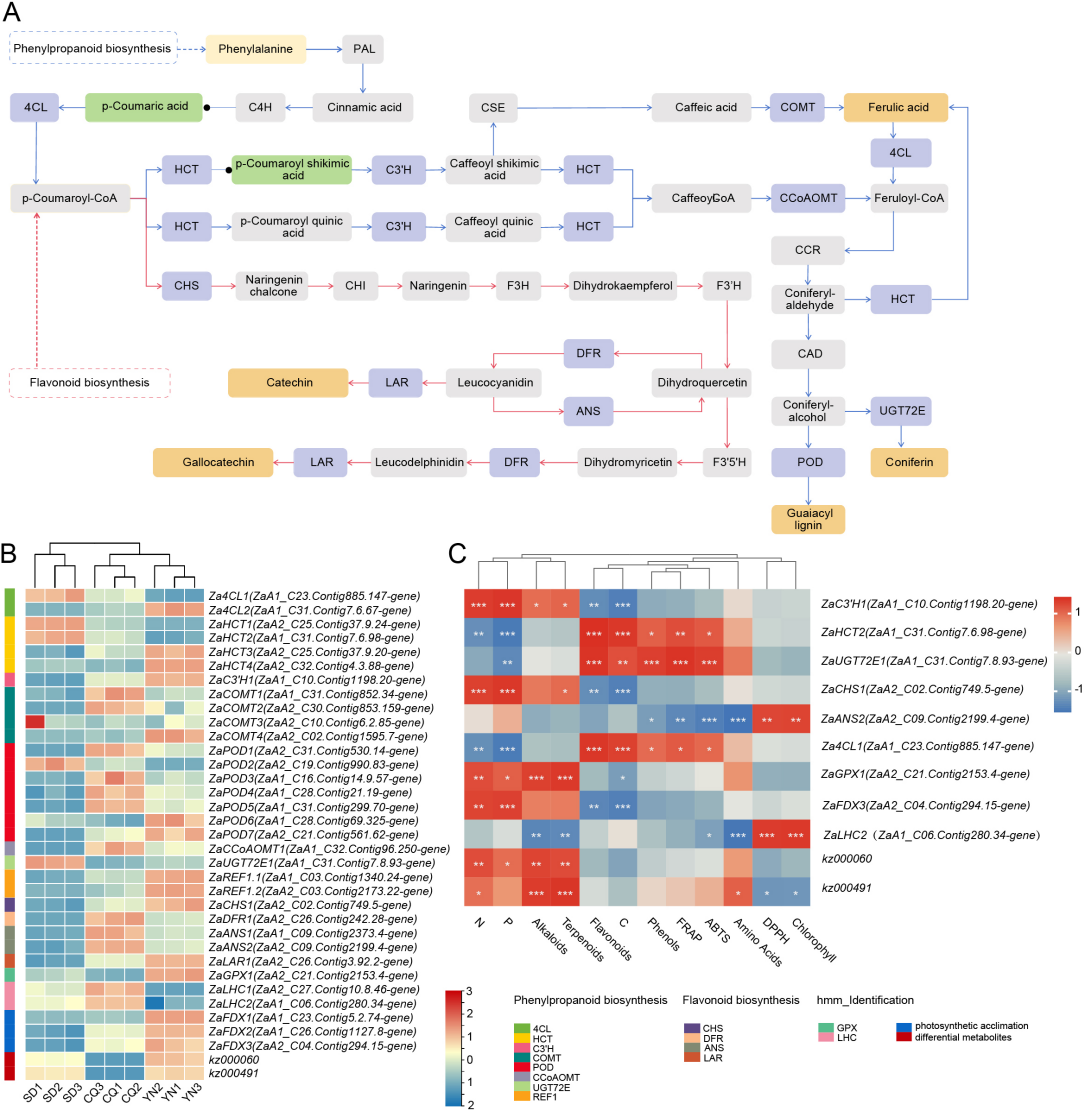
**(A)** KEGG pathway diagram highlighting the phenylpropanoid (blue) biosynthesis and flavonoid (red) biosynthesis pathways. Green boxes and black dots represent enriched metabolites, blue represents key regulatory nodes, and orange represents final products. **(B)** Heatmap displaying the expression levels of differential genes, with a color gradient from red (high expression) to blue (low expression). The scale represents log2-transformed gene expression values. **(C)** Correlation heatmap between differential genes and metabolites related to resistance and photosynthesis, and physiological indicators. The correlation values range from -1 to 1, indicating strong positive to negative correlations. Significance is marked with asterisk (*), with red and blue represent positive and negative correlations, respectively.

Within these two pathways, 27 DEGs and two DEMs—*kz000060* and *kz000491*—were mapped. These metabolites represent intermediates involved in the biosynthesis of flavan-3-ols and phenolic acids, respectively. KEGG pathway mapping was used to visualize transcript and metabolite integration (**Figure 6A**). In the phenylpropanoid pathway, *4-*coumarate-CoA ligase, *Za4CL1* (*4CL*, *ZaA1_C23.Contig885.147-gene*) catalyzes the formation of *p-*coumaroyl-CoA from *p-*coumaric acid. This intermediate is then conjugated with shikimic acid by hydroxycinnamoyl-CoA shikimate/quinate hydroxycinnamoyltransferase *ZaHCT2* (*ZaA1_C31.Contig7.6.98-gene*) to produce *trans-*5*-O-p*-coumaroyl shikimic acid (*kz000060*), followed by hydroxylation, methylation, and polymerization reactions involving *C3’H*, *COMT*, *POD*, *REF1*, and *UGT72E*. In the flavonoid pathway, *CHS*, *DFR*, *ANS*, and *LAR* catalyze the synthesis of anthocyanins and flavan-3-ols such as catechins. Expression heatmaps based on FPKM values (**Figure 6B**) revealed population-specific expression patterns across the phenylpropanoid and flavonoid biosynthesis pathways. The SD group exhibited higher expression of upstream genes but lower expression in mid- and downstream segments. The YN group showed relatively balanced and elevated expression throughout the pathway, particularly in mid- and downstream steps, suggesting greater transcriptional continuity and metabolic efficiency. The CQ group displayed overall lower expression, indicating limited activity in these pathways. These results indicate that redox-related secondary metabolic pathways are differentially regulated among the three populations, with the YN group demonstrating more coordinated and efficient gene expression.

### 3.7 Screening and functional analysis of photosynthesis-related genes

Based on observed differences in stomatal density among the three *Z. armatum* populations (**Figure 2A**), this study further examined genes potentially related to photosynthetic processes. Among the enriched GO terms, photosynthetic acclimation was identified as functionally related to light adaptation. Three DEGs annotated under this term, *ZaFDX1* (*ZaA1_C23.Contig5.2-gene*), *ZaFDX2* (*ZaA1_C26.Contig1127.8-gene*), and *ZaFDX3* (*ZaA2_C04.Contig294.15-gene*) were selected for further analysis. Pfam domain annotation revealed that these genes belong to the ferredoxin [2Fe-2S] family, a group of proteins involved in electron transport from photosystem I (PSI) to NADP^+^ reductase (FNR), thereby contributing to NADPH production in the light reactions of photosynthesis. NADPH is required for CO_2_ fixation in the Calvin cycle (Hanke and Mulo, 2013; Schorsch et al., 2018). In addition to their role in photosynthetic electron transfer, ferredoxins participate in nitrogen and sulfur metabolism, indicating broader metabolic functions.

To identify additional genes associated with light capture, a genome-wide screening of the light-harvesting complex (LHC) gene family was conducted. A total of 83 candidate LHC genes were identified (E-value < 0.05; **Supplementary Table S16**). By intersecting this list with the 1,962 DEGs, two LHC genes, *ZaLHC1* (*ZaA2_C27.Contig10.8.46-gene*) and *ZaLHC2* (*ZaA1_C06.Contig280.34-gene*) were selected for further analysis. LHC proteins bind chlorophyll to absorb light energy and transfer it to PSI and PSII reaction centers, facilitating the initiation of electron transport (Levin and Schuster, 2023; Teramoto et al., 2002). Correlation analysis showed that both LHC genes were significantly positively associated with chlorophyll content (**Figure 6C**), suggesting a potential link to light-harvesting capacity. These genes also showed correlations with terpenoid and other secondary metabolites, indicating potential associations between photosynthesis-related transcriptional activity and secondary metabolic variation.

### 3.8 Correlation and expression level analysis

Nine genes showing pronounced inter-population expression differences were selected based on expression levels, excluding those with zero or aberrant expression. These included seven resistance-related genes identified through GO enrichment, KEGG pathway analysis, and gene family annotation, and two photosynthesis-related genes obtained from GO terms and gene family screening. Additionally, two differentially expressed metabolites (*kz000060* and *kz000491*), enriched in phenylpropanoid and flavonoid biosynthesis pathways, were incorporated into subsequent correlation analyses with physiological and biochemical indicators (**Figure 6C**). Expression profiles of the selected genes revealed clear regional variation. The seven resistance-related genes—*Za4CL1* (*ZaA1_C23.Contig885.147-gene*), *ZaHCT2* (*ZaA1_C31.Contig7.6.98-gene*), *ZaC3’H1* (*ZaA1_C10.Contig1198.20-gene*), *ZaCHS1* (*ZaA2_C02.Contig749.5-gene*), *ZaANS2* (*ZaA2_C09.Contig2199.4-gene*), *ZaGPX1* (*ZaA2_C21.Contig2153.4-gene*), and *ZaUGT72E1* (*ZaA1_C31.Contig7.8.93-gene*)—displayed significantly different expression levels among the CQ, YN, and SD groups. Similarly, the two photosynthesis-related genes, *ZaFDX3* (*ZaA2_C04.Contig294.15-gene*) and *ZaLHC2* (*ZaA1_C06.Contig280.34-gene*), showed distinct region-specific expression patterns.

This study then assessed the correlations between these genes and metabolites with key physiological and biochemical indicators, including secondary metabolites and antioxidant capacity measurements (**Figure 6C**). The seven resistance-related genes showed significant correlations with levels of alkaloids, terpenoids, flavonoids, phenols, as well as with FRAP, ABTS, and DPPH values. Among the two differentially expressed metabolites, *kz000060* was significantly correlated with nitrogen (N), phosphorus (P), alkaloid, and terpenoid contents, while *kz000491* was significantly associated with N, alkaloids, terpenoids, and amino acid levels. These associations suggest a potential role of these metabolites in nutritional regulation and defense-related metabolism. Regarding the two photosynthesis-related genes, *ZaFDX3* (*ZaA2_C04.Contig294.15-gene*) was positively correlated with N and P content, two essential macronutrients involved in chlorophyll synthesis and photosynthetic metabolism. *ZaLHC2* (*ZaA1_C06.Contig280.34-gene*) showed strong significant positive correlations with chlorophyll content and DPPH activity. This association may reflect a coordinated relationship between antioxidant status and photosynthetic metabolism.

### 3.9 qRT-PCR validation

**Figure 7:**
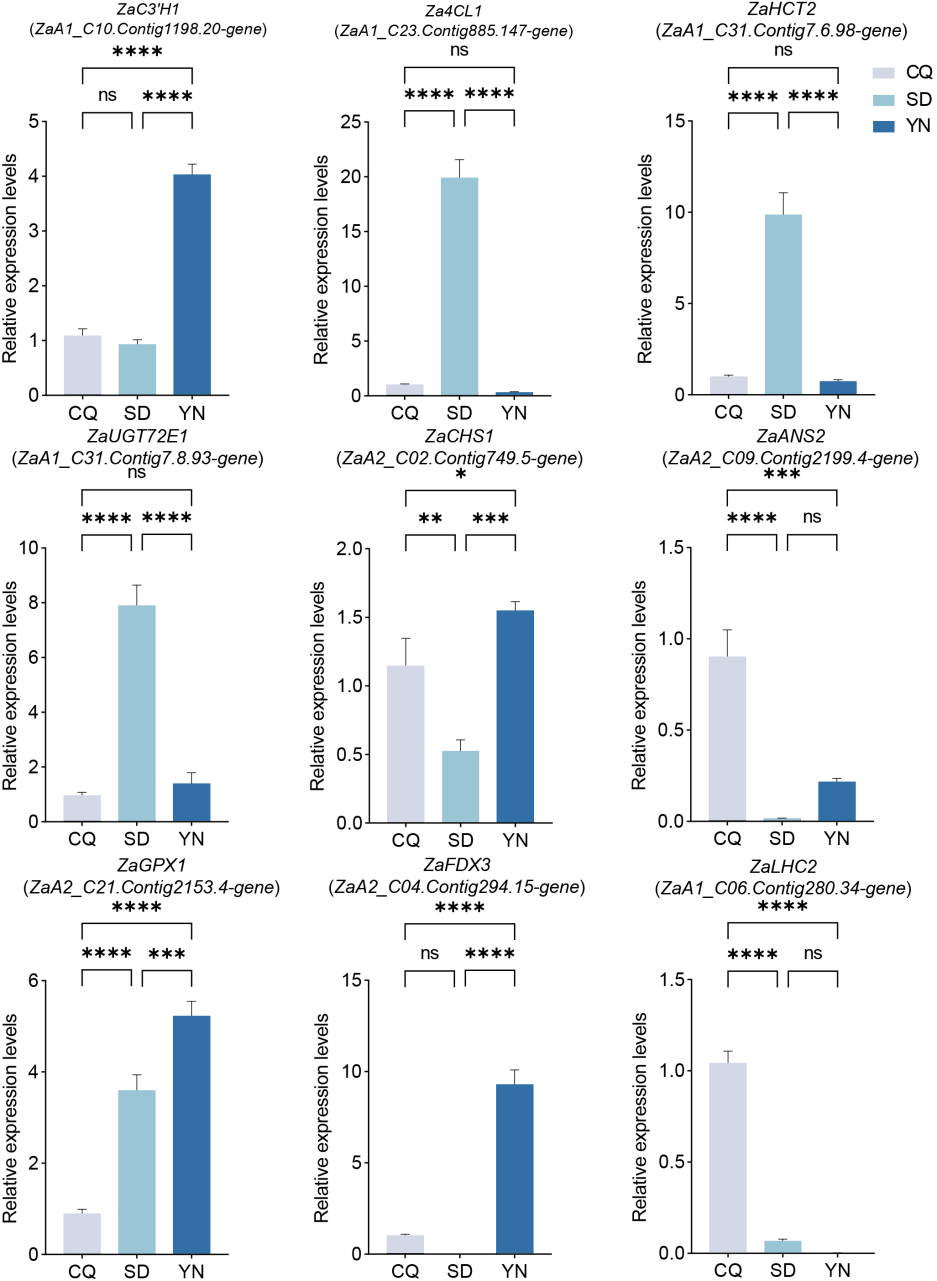
Expression levels of seven differentially expressed genes related to resistance and two differentially expressed related to photosynthesis. CQ is represented by gray, YN by dark blue, and SD by light blue. ‘ns’ indicates non-significant, * indicates *P* < 0.05, ** indicates *P* < 0.01, *** indicates *P* < 0.001, and **** indicates *P* < 0.0001.

To validate the reliability of the RNA-seq data, we performed qPCR validation on seven resistance-related genes and two photosynthesis-related genes previously selected based on their differential expression profiles (**Table 1**). Data were visualized using GraphPad Prism 9 (**Figure 7**). The results showed a high degree of consistency between the qPCR and RNA-seq gene expression trends, indicating the high reliability of the RNA-seq data. For example, *ZaC3’H1* (*ZaA1_C10.Contig1198.20-gene*) showed a medium-low-high trend across CQ, SD, and YN in qPCR validation, and *ZaGPX1* (*ZaA2_C21.Contig2153.4-gene*) showed a low-to-high trend, whereas *ZaLHC2* (*ZaA1_C06.Contig280.34-gene*) showed a high-to-low trend, all of which aligned with the RNA-seq FPKM value trends. Although the overall trends were consistent, some genes did not exhibit significant differences in expression in certain regions. Among the photosynthesis-related genes, *ZaLHC2* (*ZaA1_C06.Contig280.34-gene*) showed significant differences in CQ, SD, and YN, despite its very low expression in the YN group (**Figure 7**). Similarly, *ZaFDX3* (*ZaA2_C04.Contig294.15-gene*) showed very low expression in the SD group.

**Table 1:** The information of qRT-PCR primers.

Gene name	F (Primer sequence 5’-3’)	R (Primer sequence 5’-3’)
UBQ	TCGAAGATGGCCGTACATTG	TCCTCTAAGCCTCAGCACCA
*ZaC3’H1* (*ZaA1_C10.Contig1198.20-gene*)	CCACCAACACCTCTAATGCT	CGTCCTCTTCGATGAACCTC
*ZaHCT2* (*ZaA1_C31.Contig7.6.98-gene*)	TGTGTATTTCTATCGTCGCC	TCCAAAAACAAAACTCCCTC
*ZaUGT72E1* (*ZaA1_C31.Contig7.8.93-gene*)	GCCCCTGTTTATGCAGTTGG	GTTTTGATGATAGCGTCCCG
*ZaCHS1* (*ZaA2_C02.Contig749.5-gene*)	GTCAAGACATTGTGGTGGT	GAGTTTGGTGAGCTGGTAG
*ZaANS2* (*ZaA2_C09.Contig2199.4-gene*)	AGGATTAGAAGTGGGGAGG	TGGAACCATGTTGTGAAGA
*Za4CL1* (*ZaA1_C23.Contig885.147-gene*)	GAAGCCCAAGTAGTCAGTGT	ATATATCCAAGATCTCCCGT
*ZaGPX1* (*ZaA2_C21.Contig2153.4-gene*)	GATAGCAGAGGCAAATAC	ATCAGTCAACTGGGAGTA
*ZaFDX3* (*ZaA2_C04.Contig294.15-gene*)	GCTGGTTCTTGCTCTTCT	CTTCTTCCTTGTGGGTCT
*ZaLHC2* (*ZaA1_C06.Contig280.34-gene*)	GGCAGTGGAGCTATCCAA	CACGCTGACACCTTTGAA

## 4 Discussion

### 4.1 Multi-level differentiation reveals regional adaptation strategies in *Z. armatum*

*Z. armatum* is an important economic and ecological tree species (Ye et al., 2022), and understanding the adaptive divergence across geographic origins is critical for its germplasm utilization and sustainable cultivation. This study demonstrated that even under common garden, individuals from three latitudinal origins (SD, CQ, YN) maintained stable phenotypic, physiological, and molecular differences, reflecting distinct environmental response strategies shaped by long-term ecological backgrounds (**Supplementary Figure S2**).

The SD group, originating from a high-latitude, cold-dry region with abundant sunlight, exhibited compact leaf morphology, high stomatal density, and elevated flavonoid and phenol accumulation. These traits suggest improved tolerance to low temperature and limited water availability through enhanced gas exchange and antioxidant capacity (Andrade et al., 2022; Karavolias et al., 2023; Adhikari et al., 2023; Khan et al., 2021). At the transcriptional level, the SD group exhibited a greater frequency of alternative splicing events and a higher number of detected SNPs, which reflects active transcriptome remodeling and potential flexibility in gene regulation (Zhong et al., 2024; Zhang et al., 2017; Deb et al., 2023). However, this group also showed limited expression of midstream and downstream metabolic genes, indicating less efficient pathway integration and reduced overall metabolic flux.

In contrast, the CQ group from a humid, high-temperature, and low-light region displayed moderate leaf size, stomatal density, and high chlorophyll content. These traits likely represent a balanced strategy for maintaining photosynthesis under low light and high humidity (Croft et al., 2017; Wang et al., 2022). While mid- and downstream gene expression was active, limited upstream activity may constrain overall metabolic efficiency. The lower SNP frequency suggests a conservative regulatory mechanism favoring environmental stability over plasticity.

The climate in YN, with mild temperatures, abundant rainfall, and ample sunlight, provides rich resources, particularly nitrogen and phosphorus, supporting robust plant growth. *Z. armatum* from the YN group exhibited larger leaves, higher nitrogen and phosphorus content, and accumulated alkaloids and terpenoids, indicating a resource-based growth and defense strategy consistent with previous findings (Plett et al., 2021; Kang et al., 2023). Moderate stomatal density ensured efficient photosynthesis and reduced water loss, contributing to balanced resource utilization. High expression levels of genes across metabolic pathways supported efficient metabolic flux conversion, enhancing the capacity to respond to environmental variation (Nomura et al., 2018). It is noteworthy that the YN group exhibited relatively low SNP frequency and high genomic stability, yet demonstrated higher metabolic flux efficiency. This observation suggests that effective flux regulation is not solely determined by the abundance or diversity of genetic variation, but is also influenced by multiple factors such as enzyme kinetics, metabolite concentrations, and resource allocation patterns within metabolic networks (Tong et al., 2021). Therefore, the YN group may achieve strong metabolic performance through optimized expression coordination and pathway integration, without relying on mutation-induced variability. In addition, its lower mutation rate and higher genomic stability may reflect a long-term, stable growth strategy that supports greater resilience under varying environmental conditions (Cai et al., 2016; Dai et al., 2024).

### 4.2 Molecular mechanisms underlying stress resistance and photosynthetic regulation

Key genes and metabolites play a crucial role in regulating traits associated with population-level variation, including resistance and photosynthesis in *Z. armatum*., *Za4CL1* (*ZaA1_C23.Contig885.147-gene*) (PF13193, PF00501) (Nie et al., 2023), *ZaHCT2* (*ZaA1_C31.Contig7.6.98-gene*) (PF02458) (Xu et al., 2023a), and *ZaC3’H1* (*ZaA1_C10.Contig1198.20-gene*) (PF00067) (Ehlting et al., 2006), along with *ZaUGT72E1* (*ZaA1_C31.Contig7.8.93-gene*) (PF00201) (Dong et al., 2020) are involved in lignin and phenolic compound biosynthesis, contributing to structural integrity and defense-related metabolism. *ZaCHS1* (*ZaA2_C02.Contig749.5-gene*) (PF00195, PF02797) (Xie et al., 2025) and *ZaANS2* (*ZaA2_C09.Contig2199.4-gene*) (PF03171, PF14226) (Chauhan et al., 2023) participate in flavan-3-ol biosynthesis and antioxidant regulation via ROS scavenging, while *ZaGPX1* (*ZaA2_C21.Contig2153.4-gene*), belonging to the GPX gene family, alleviates oxidative stress, thereby effectively protecting plants from environmental stress damage (Jiang et al., 2023). Furthermore, these genes work synergistically with differential metabolites such as *trans-*5-*O*-*p*-coumaroyl shikimic acid and *p-*coumaric acid, further enhancing plant stress resistance and adaptability. In terms of photosynthesis regulation, *ZaFDX3* (*ZaA2_C04.Contig294.15-gene*) (PF00111) and *ZaLHC2* (*ZaA1_C06.Contig280.34-gene*) (LHC family, PF00504), which were significantly positively correlated with chlorophyll, play an important role in the light reaction process. *ZaFDX3* (*ZaA2_C04.Contig294.15-gene*) is involved in regulating ferredoxin, whereas *ZaLHC2* (*ZaA1_C06.Contig280.34-gene*) regulates LHC proteins, enhancing light energy capture and energy conversion efficiency. Additionally, qPCR validation of the expression of the selected differential genes showed that the expression trends were highly consistent with the RNA-seq data (**Figure 7**), further supporting the reliability of the transcriptomic data.

This study identified seven resistance-associated and two photosynthesis-related genes that were differentially expressed among *Z. armatum* populations grow in the common garden conditions. Together with associated metabolites, these genes may contribute to population-level variation in antioxidant regulation and photosynthetic efficiency through coordinated gene expression and metabolic pathway integration. Such differences likely reflect the combined effects of physiological traits, transcriptomic profiles, and metabolic activity, independent of immediate environmental factors. In the future, using these key genes as targets, CRISPR-Cas9, RNA interference, and artificial microRNA technologies could be combined to decipher the roles of these genes in ecological niche adaptation and explore their potential in coordinating secondary metabolism, thereby synergistically enhancing both the edible and medicinal properties of the plant. This will provide new theoretical foundations and technical support for the development of superior germplasm resources, the breeding of new breakthrough varieties, and sustainable development.

## Data Availability

The original contributions presented in the study are publicly available. The RNA-seq data have been deposited in the National Genomics Data Center (https://ngdc.cncb.ac.cn/), BioProject accession PRJCA037036.

## Supplementary material

Supplementary Figure 1 OPLS-DA model validation via permutation tests.

Supplementary Figure 2 Illustration of the graphical abstract.

Supplementary Figure 3 Validation of reference gene stability.
