# Supplementary figures and images for: Integrated morphophysiological, transcriptomic, and metabolomic data uncover the molecular mechanism of environmental adaptation of Zanthoxylum armatum with different latitudinal gradients
Source: Front Plant Sci. 2025 Jul 1;16:1622956. doi: 10.3389/fpls.2025.1622956 (PMC12259641; doi:10.3389/fpls.2025.1622956)

CQ\_SD

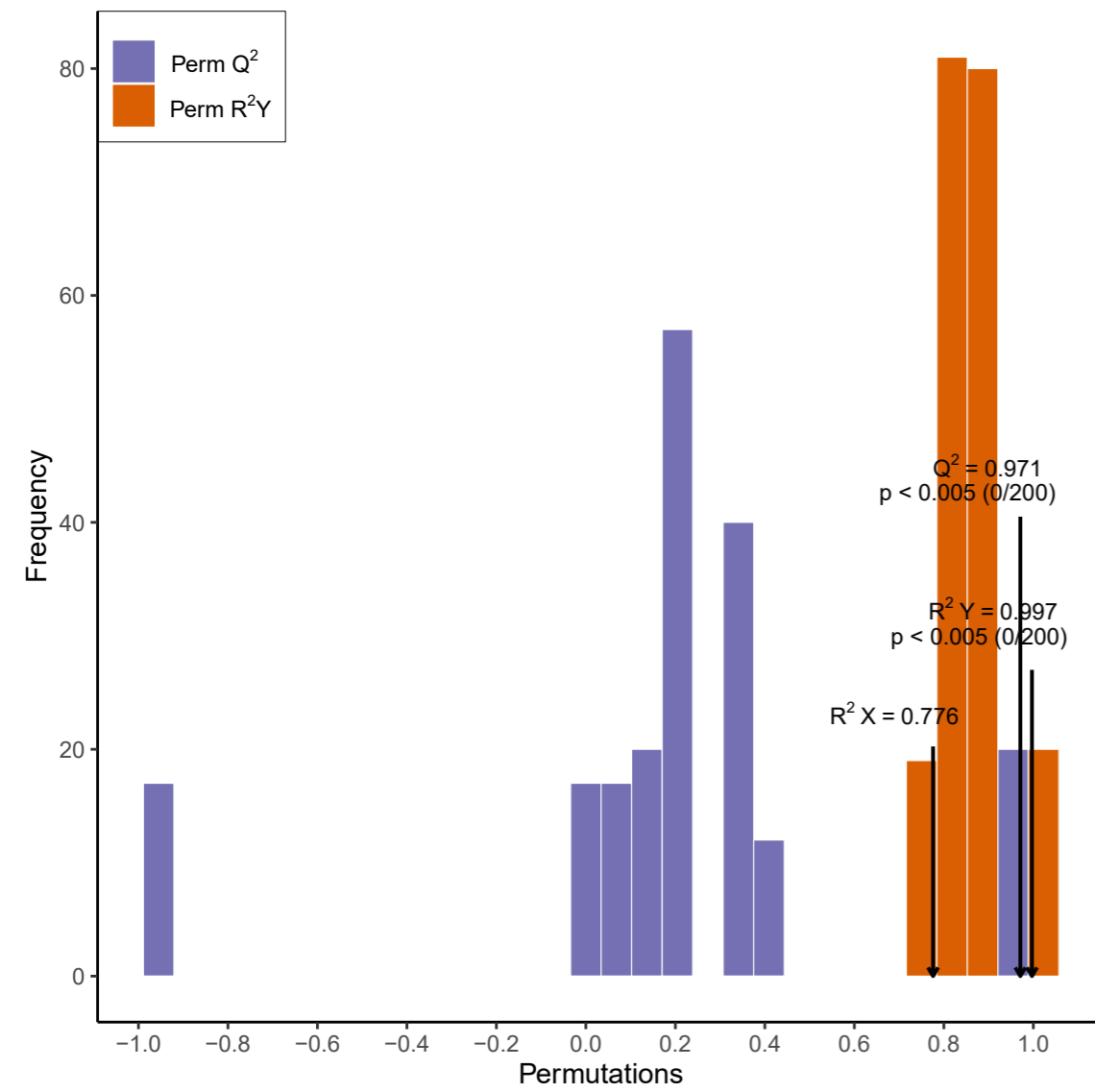

CQ\_YN

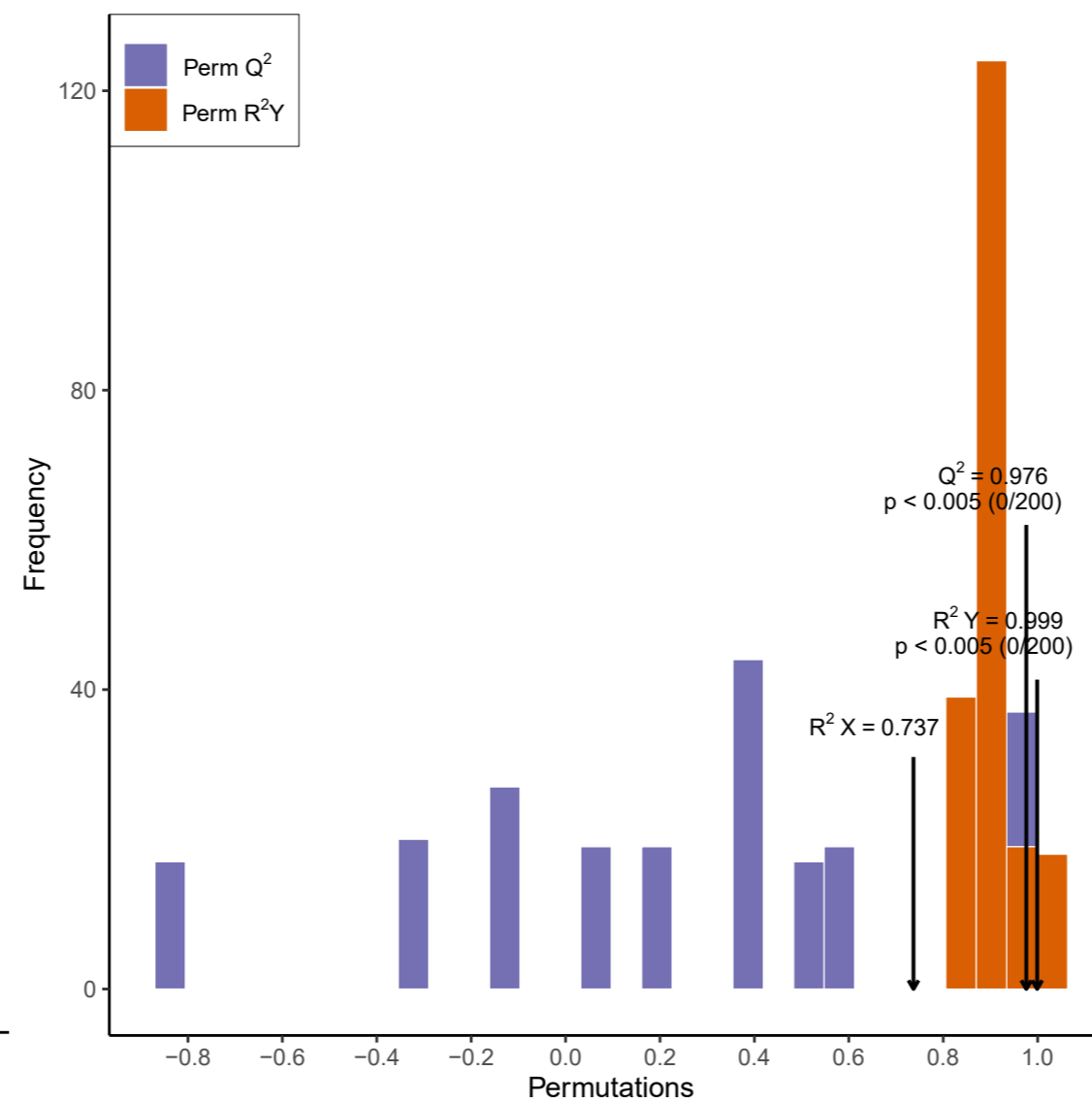

SD\_YN

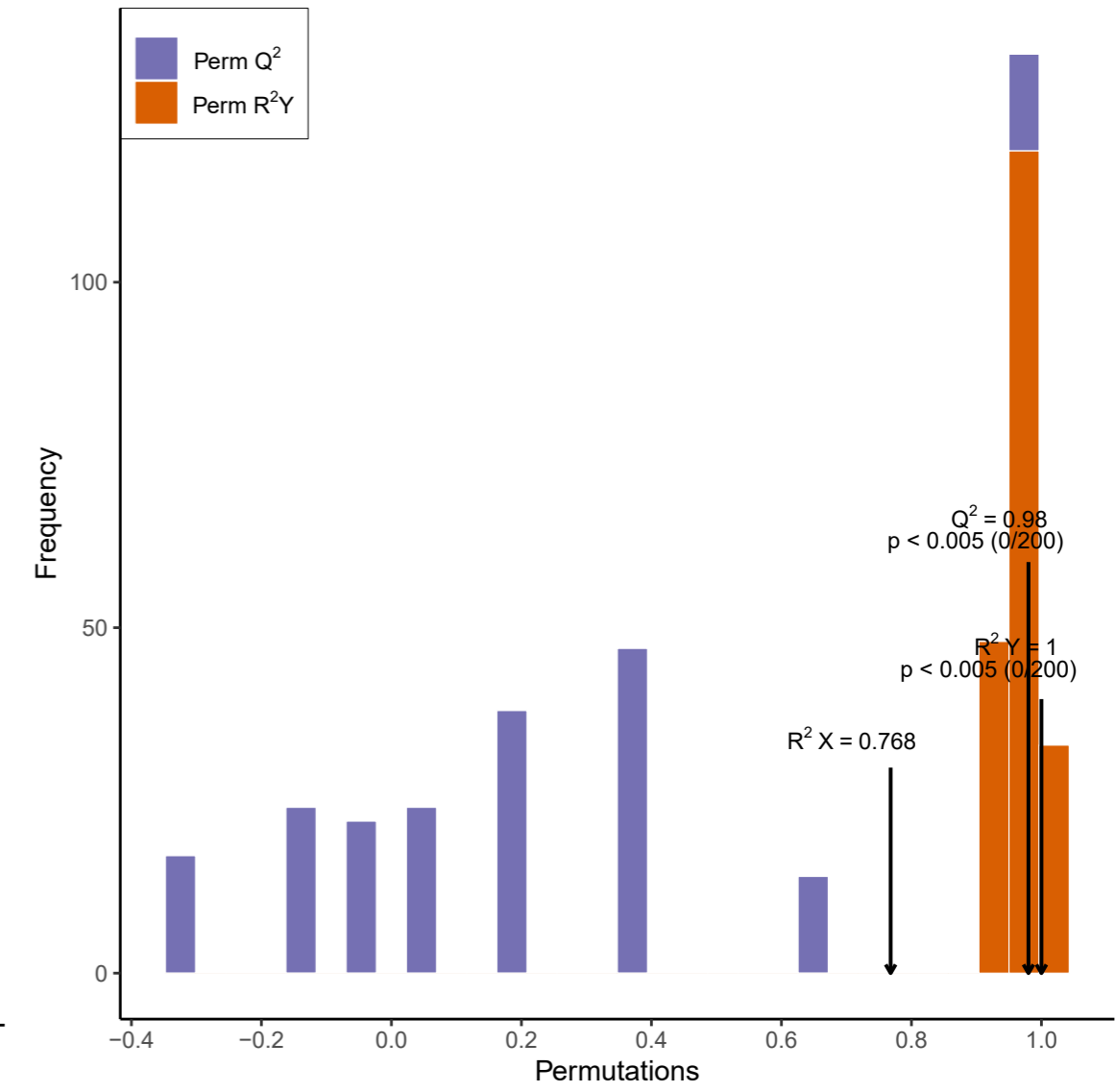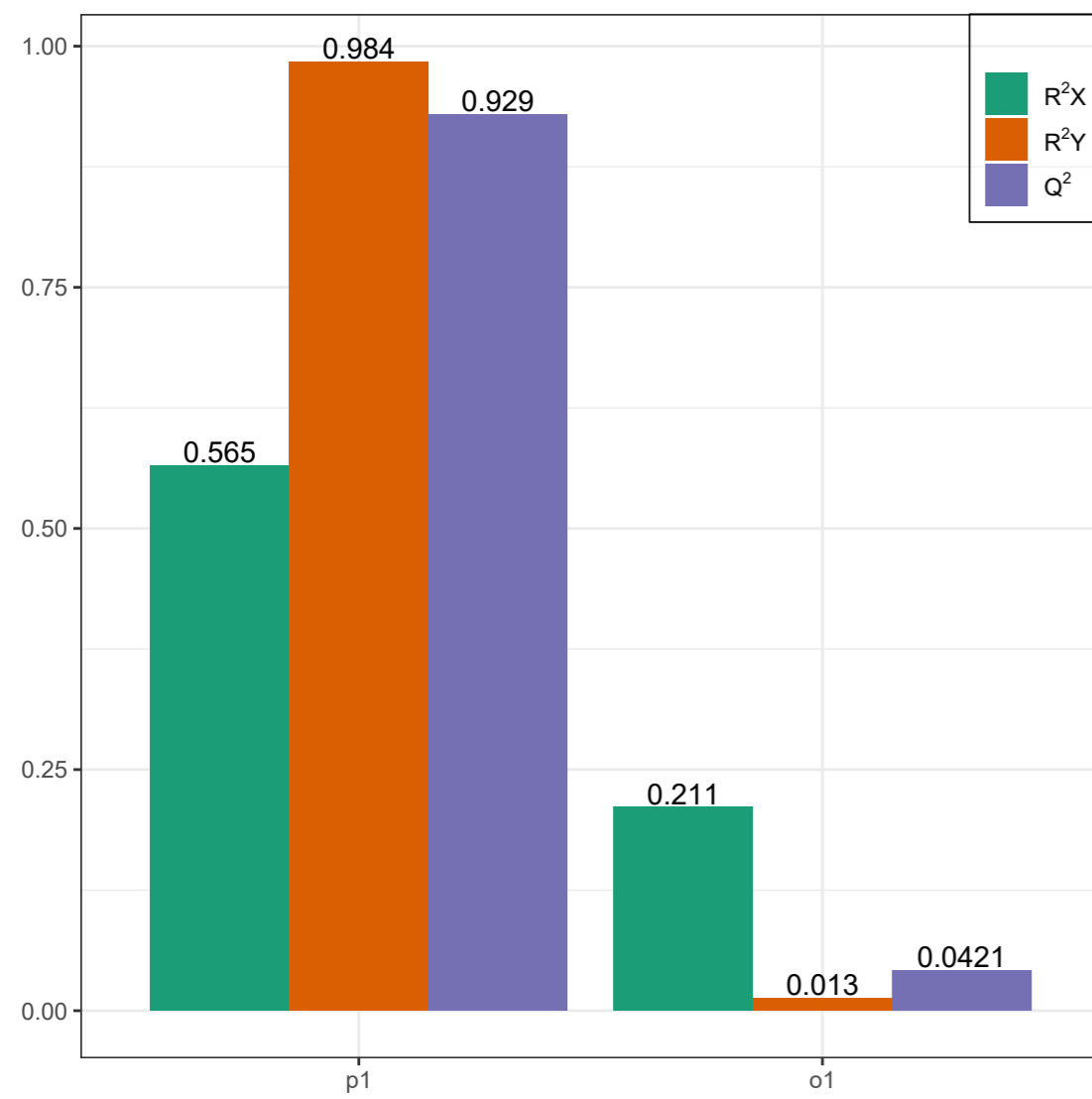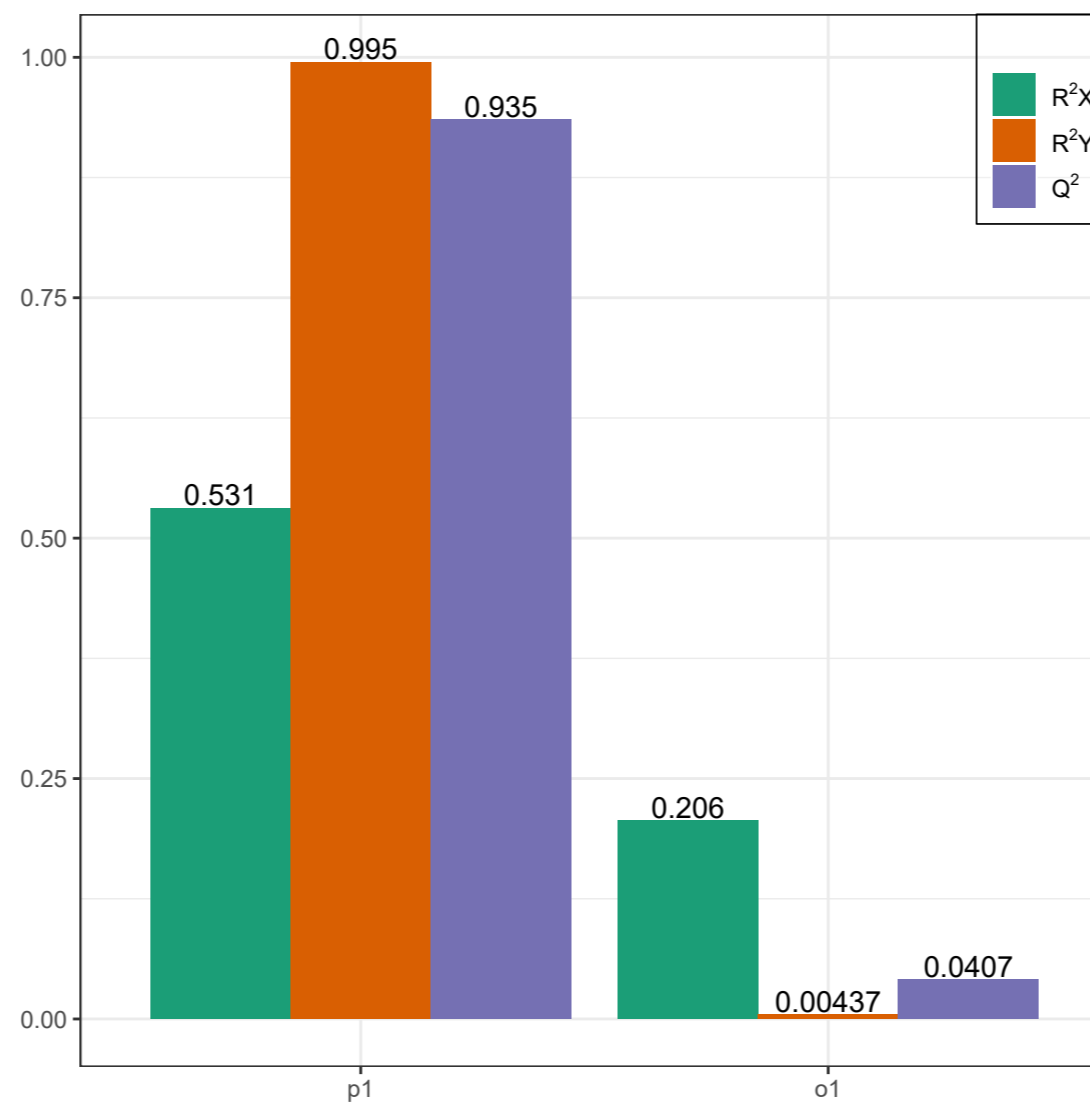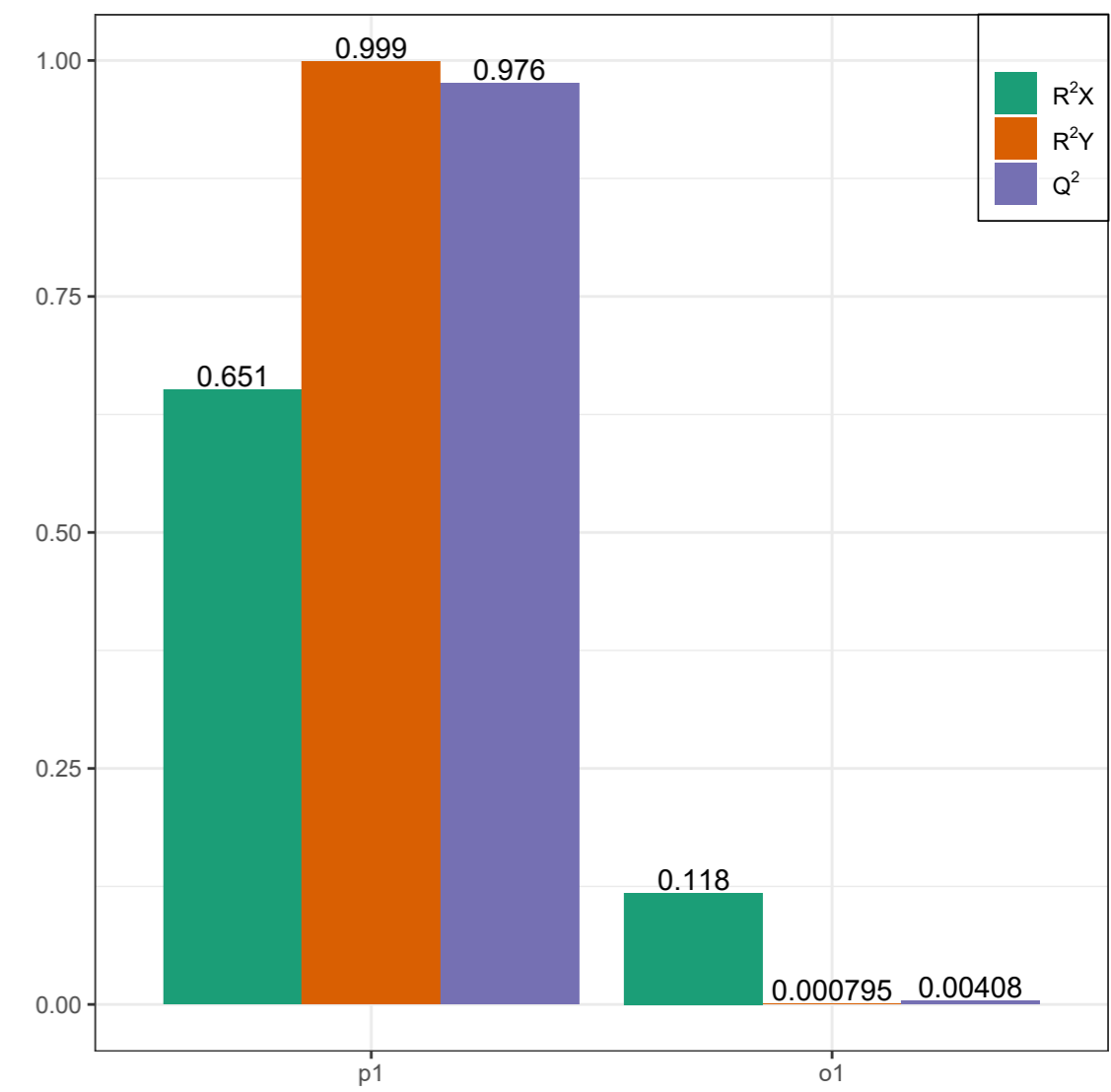

Supplement: Supplementary Figure 1 — OPLS-DA model validation via permutation tests. [file Image1.pdf]

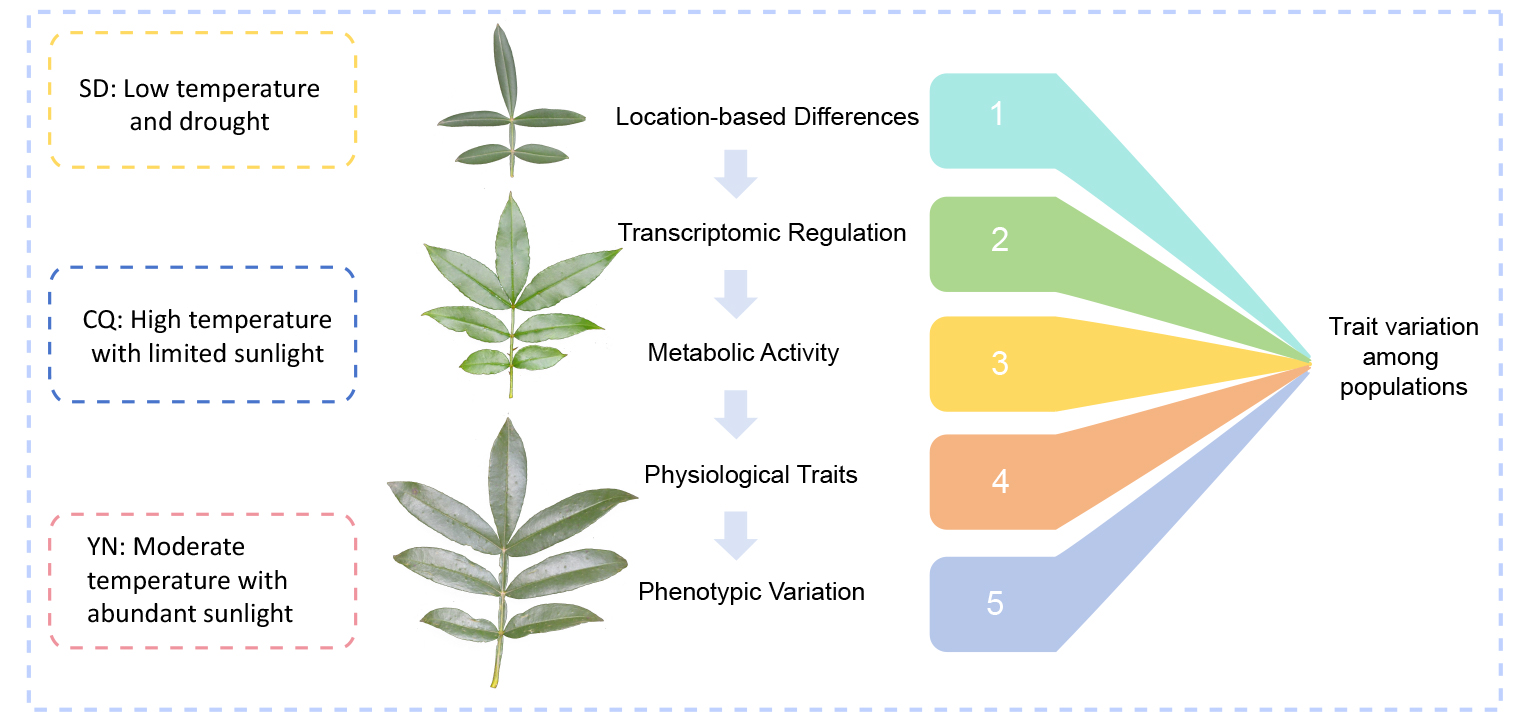

Supplement: Supplementary Figure 2 — Illustration of the graphical abstract. [file Image2.jpeg]

Gene stability by Genorm

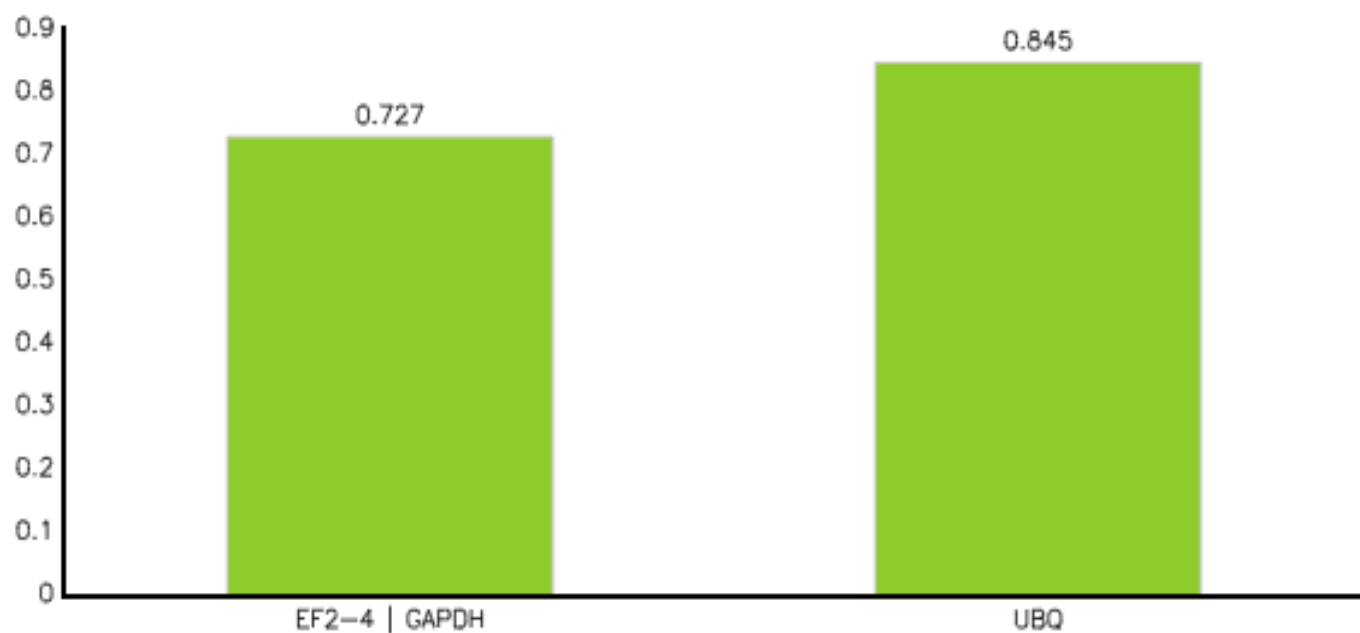

Gene stability by BestKeeper

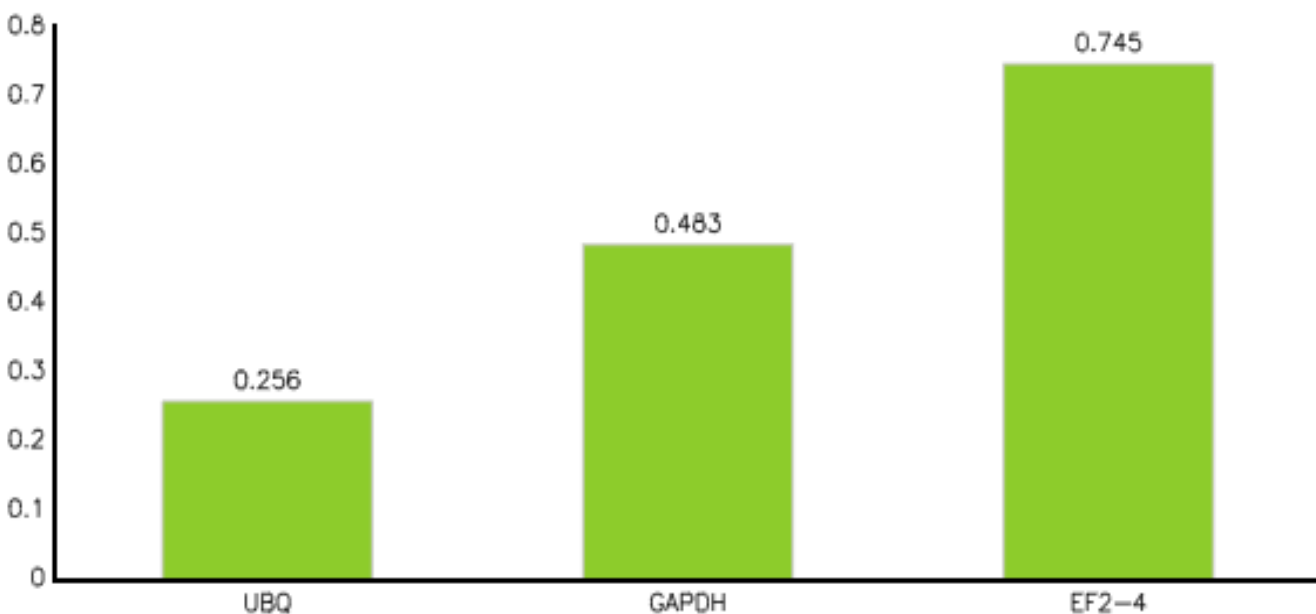

Supplement: Supplementary Figure 3 — Validation of reference gene stability. [file Image3.pdf]
